# Identifying Markers of Emerging SARS-CoV-2 Variants in Patients With Secondary Immunodeficiency

**DOI:** 10.3389/fmicb.2022.933983

**Published:** 2022-07-01

**Authors:** Nathan M. Markarian, Gaël Galli, Dhanesh Patel, Mark Hemmings, Priya Nagpal, Albert M. Berghuis, Levon Abrahamyan, Silvia M. Vidal

**Affiliations:** ^1^Department of Human Genetics, McGill University, Montréal, QC, Canada; ^2^McGill University Research Centre on Complex Traits, Montréal, QC, Canada; ^3^Swine and Poultry Infectious Diseases Research Center and Research Group on Infectious Diseases in Production Animals, Faculty of Veterinary Medicine, University of Montreal, Saint-Hyacinthe, QC, Canada; ^4^Department of Microbiology and Immunology, McGill University, Montréal, QC, Canada; ^5^CNRS, ImmunoConcEpT, UMR 5164, Université de Bordeaux, Bordeaux, France; ^6^CHU de Bordeaux, FHU ACRONIM, Centre National de Référence des Maladies Auto-Immunes et Systémiques Rares Est/Sud-Ouest, Bordeaux, France; ^7^Department of Biochemistry, McGill University, Montréal, QC, Canada; ^8^Department of Pharmacology, McGill University, Montréal, QC, Canada

**Keywords:** SARS-CoV-2, viral evolution, secondary immunodeficiency, mutations, spike protein, COVID-19

## Abstract

Since the end of 2019, the world has been challenged by the coronavirus disease 2019 (COVID-19) pandemic. With COVID-19 cases rising globally, severe acute respiratory syndrome coronavirus 2 (SARS-CoV-2) continues to evolve, resulting in the emergence of variants of interest (VOI) and of concern (VOC). Of the hundreds of millions infected, immunodeficient patients are one of the vulnerable cohorts that are most susceptible to this virus. These individuals include those with preexisting health conditions and/or those undergoing immunosuppressive treatment (secondary immunodeficiency). In these cases, several researchers have reported chronic infections in the presence of anti-COVID-19 treatments that may potentially lead to the evolution of the virus within the host. Such variations occurred in a variety of viral proteins, including key structural ones involved in pathogenesis such as spike proteins. Tracking and comparing such mutations with those arisen in the general population may provide information about functional sites within the SARS-CoV-2 genome. In this study, we reviewed the current literature regarding the specific features of SARS-CoV-2 evolution in immunocompromised patients and identified recurrent *de novo* amino acid changes in virus isolates of these patients that can potentially play an important role in SARS-CoV-2 pathogenesis and evolution.

## Introduction

In late 2019, a new viral outbreak in Wuhan city, China (World Health Organization [WHO], 2020a), rapidly identified as the severe acute respiratory syndrome coronavirus 2 (SARS-CoV-2), resulted in the coronavirus disease 2019 (COVID-19) pandemic (World Health Organization [WHO], 2020b), which still continues with the rise of novel variants of concern (VOCs) and of interest (VOIs).

Increased age is perhaps the strongest risk factor for severe COVID-19 (Bonanad et al., 2020); obesity, male gender, and various comorbidities such as hypertension, cardiovascular disease, and diabetes also contribute to an increased odds ratio of severe disease (Hu and Wang, 2021). However, among infected individuals, patients with secondary immunodeficiency, due to preexisting health conditions, and those undergoing immunosuppressive treatment are particularly susceptible to SARS-CoV-2 (Gao et al., 2020a; Liu and Hill, 2020; Hoffmann et al., 2021; Jones et al., 2021). Many research groups have reported chronic infections and the accumulation of viral protein-coding mutations in such individuals in the presence of anti-COVID-19 treatments, with potential relevance at both biological and epidemiological levels. We hypothesized that two main kinds of mutations could be observed in such immunodeficient setting, namely, (1) variations selected by antiviral treatment and (2) variations reflecting the adaptation of the virus to the human host, particularly in the context of an environment with reduced immune responses, allowing niches of selective pressure.

To gain insights into the mutational signatures of secondary immunodeficiency in SARS-CoV-2 genetic profiles, we have queried the literature to review SARS-CoV-2 genome data from 44 patients with secondary immunodeficiency who underwent treatment against COVID-19. We retrieved 148 full genomes from 21 patients and partial genomes for 24 patients. By analyzing the viral genomes detected in these patients in comparison with circulating variants, we identified numerous new protein-coding mutations and inspected their predicted structural or functional impact at the protein level.

## SARS-CoV-2

**FIGURE 1:**
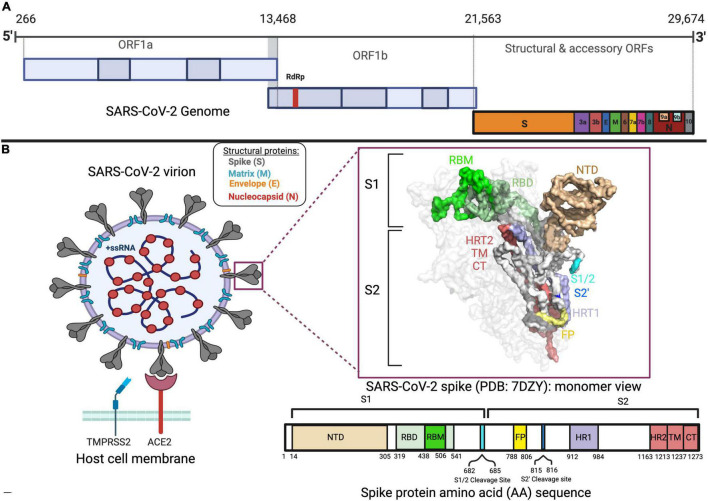
SARS-CoV-2 genome, spike, and virion. **(A)** The genomic sequence of SARS-CoV-2 with different open reading frames (ORFs) is displayed in different colors. **(B)** Representation of the SARS-CoV-2 virion structure, spike protein and amino acid sequence of spike protein, and its domains in different colors. The binding of SARS-CoV-2 virion to the ACE2 receptor adjacent to the TMPRSS2 protein is also shown.

SARS-CoV-2 is a betacoronavirus that shares 96% of its genomic identity with the RaTG13 bat coronavirus and is hypothesized to be of zoonotic origin (Zhou et al., 2020; Banerjee et al., 2021). It is a positive sense ribonucleic acid virus (RNA), with a genome spanning around 30 kilobases in length (Wu et al., 2020; V’kovski et al., 2021). Notably, two-thirds of its genome is composed of overlapping open reading frames (ORF) 1a and 1b, which together encode for an RNA-dependent RNA polymerase (RdRp) and other non-structural proteins important for viral replication and transcription (Figure 1A; Wang Q. et al., 2020; Wang Y. et al., 2020; Yan et al., 2021). The remainder of the viral genome is composed of ORFs 2–10 encoding for structural and accessory proteins (Figure 1A; Davidson et al., 2020; Jiang et al., 2020; Michel et al., 2020; Mohammad et al., 2020; Pancer et al., 2020).

Of the structural proteins, the spike is a large accessible homotrimeric protein of great importance in viral tropism and viral entry, making it a great target in therapeutic development (Conceicao et al., 2020). With a molecular weight of around 180 kDa, the spike protein is composed of 2 major subunits per monomer: the S1 (residues 14–685) and S2 (residues 686–1273) (Figure 1B; Huang et al., 2020; Martí et al., 2021). The former is the most variable part of the spike among coronaviruses and contains the amino (N)-terminal domain (NTD) and the receptor-binding domain (RBD) (Figure 1B; Huang et al., 2020; Martí et al., 2021). As for the S2, its domains, which are essential for viral fusion with the host cell membrane, are more conserved in structure and sequence (Figure 1B; Huang et al., 2020; Martí et al., 2021).

*The main target of the spike is* the angiotensin-converting enzyme (ACE2) (Li et al., 2020). The broad expression of ACE2 explains in part SARS-CoV-2 pathogenesis in a multitude of organs from respiratory, circulatory, urogenital, gastrointestinal, and nervous systems (Lopes-Pacheco et al., 2021).

*Following cell entry*, the replication of SARS-CoV-2 takes place in the cytoplasm with the help of the host ribosomal machinery, translating the ORF 1a and 1b genes into two large replicase polyproteins, namely, pp1a and pp1ab (V’kovski et al., 2021). Together, both pp1a and pp1ab polyproteins undergo proteolytic cleavages *via* the viral-encoded proteinases papain-like protease (PL-pro, Nsp3) and 3C-like protease (3CL-pro, Nsp5) to generate 16 mature non-structural proteins, i.e., Nsp1 to Nsp16 (Astuti and Ysrafil, 2020). Proteolysis is an essential step for viral replication, which is why antivirals targeting proteases are of interest (Dampalla et al., 2021; Roe et al., 2021). Later, the RNA-dependent RNA polymerase (RdRp and Nsp12), helicase (Nsp13), and Nsp7 to Nsp9 form the replication/transcription complex (RTC), allowing the synthesis of viral RNA in double-membrane vesicles (DMV) at the periphery of the endoplasmic reticulum (Brant et al., 2021).

## Mutations in Emerging Variants

**FIGURE 2:**
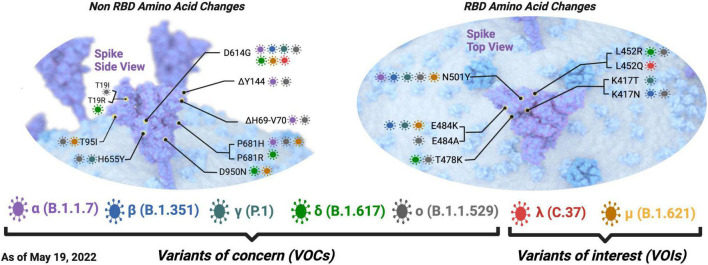
Defining spike amino acid changes in SARS-CoV-2 variant of concerns (VOCs) and interest (VOIs). The variants of concern are depicted in purple, blue, dark green, pale green, and pale gray for the alpha(α), beta (β), gamma (γ), delta (δ), and omicron (o) variants, respectively. The variants of interest are depicted in red and dark yellow for the lambda (λ) and mu (μ) variants, respectively. The envelope is shown in blue, and the spike protein is shown in purple. Common amino acid changes in different variants are also depicted. Non-RBD amino acid changes are shown on the left, and RBD amino acid changes are shown on the right. Defining amino acid changes are those appearing at the phylogenetic root of a variant (Hodcroft, 2021). Adapted from “The SARS-CoV-2 Variants of Concern,” by BioRender.com (2021).

Like most RNA viruses, SARS-CoV-2 continues to mutate as it spreads, resulting in different variants, where the Pango numeric system assigns lineages with a number or letter such as B.1 (Oude Munnink et al., 2021). Among the variants circulating as of May 08 2022, five are known VOCs defined by the WHO based on their epidemiology and their association with disease severity or potential to escape available treatments or vaccines (Figure 2; Harvey et al., 2021; World Health Organization [WHO], 2021). Martin et al. (2021) reported that there has been a shift in the mutational landscape of some VOCs with the N501Y spike amino acid substitution (alpha, beta, and gamma) where there have been mutations arising independently and repeatedly in different viral lineages at 29 genome sites from 15 March 2021 to 1 June 2021. Such converging evolution in these sites could likely occur in variants of the same and different lineages (Martin et al., 2021). Variations in spike proteins that define VOCs are highlighted in Figure 2.

As a nidovirus, SARS-CoV-2 encodes a unique proofreading enzyme 3′ to 5′ exonuclease (ExoN) involved in excising faulty nucleotides inserted by RNA polymerases, thus ensuring replication fidelity (Shannon et al., 2020; Gribble et al., 2021). Despite this proofreading mechanism, SARS-CoV-2 has shown a capacity to accumulate a wide range and high number of mutations (Chen et al., 2020). A study of samples from the first wave and second wave of COVID-19 in Japan noted a mutation rate of 1.16–1.87 × 10^–3^ base substitutions/site/year (Ko et al., 2021). This is relatively low compared with the human immunodeficiency virus (HIV) subtype B, which can have a nucleotide substitution rate ranging from 5.25 × 10^–3^ to 1.60 × 10^–2^ substitutions/site/year in gag and env-gp120 genes (Dapp et al., 2017).

However, there are more ways of generating genetic diversity; viral recombination, which is the generation of new progeny from two distinct strains of virus co-infecting a cell, is a way to generate viral genetic variation (Simon-Loriere and Holmes, 2011). In the case of SARS-CoV-2, Pollet et al. (2021) performed a recombination analysis of a variety of coronaviral sequences including 100,000 SARS-CoV-2 sequences. Through this analysis, they showed eight SARS-CoV-2 recombination events, two of them in the spike gene (Pollet et al., 2021). Earlier in 2021, a SARS-CoV-2 co-infection event of a single patient was reported with two strains with distinct lineages, which raises concern for the recombination of SARS-CoV-2 evolution (Francisco et al., 2021).

Furthermore, the genetic variability of viruses is shaped through the selection pressure of their host cell or environment. The host has multiple immune defense mechanisms at cellular, tissue, and systemic levels that can interfere with viral replication and spread. Letko et al. (2018) showed an example of MERS-CoV causing observable cytopathic effect due to the accumulation of amino acid variations in the spike protein after eight viral passages in BHK cells expressing the bat DPP4 receptor. Antiviral treatments that target specific viral proteins are another selective pressure that can result in the development of treatment-resistant mutants. For instance, the emergence of two mutations in the RdRp of murine hepatitis virus (MHV) conferred a 5.6-fold increased resistance to remdesivir (based on EC_50_ values) (Agostini Maria et al., 2018). The study of virus sequences that emerge in chronically infected patients could reveal regions of the virus genome that will be important as we prepare for and predict future variants.

## Secondary Immunodeficiencies

The most common cause of immunodeficiency is acquired immunodeficiency, meaning impaired immune response secondary to a condition or its treatment. This review will focus on the four main types we have found to be associated with COVID-19, namely, cancer, organ transplantation, HIV infection/AIDS, and autoimmune diseases.

Indeed, it has been documented that immunosuppression leads to poorer prognosis in hospitalized patients (Ponsford et al., 2021), especially in cancer and organ-transplanted patients (Elkrief et al., 2020; Bhogal et al., 2021; Coll et al., 2021), as well as in HIV-infected patients (Suwanwongse and Shabarek, 2020; Kanwugu and Adadi, 2021). In cancer, this impaired immune response can result from the medical condition itself, for example, impaired humoral response in a chronic lymphocytic leukemia or bone marrow infiltration by an acute leukemia preventing the development of normal leukocytes. But immunosuppression can also be induced by the malignancy treatment: hypogammaglobulinemia induced by B cell depletion after rituximab use or by alkylating agent that impairs DNA from replicating cells (including cancer cells and leukocytes). The same kind of treatment-induced immune impairment happens in organ transplantation contexts, with the immunosuppressive regimen used for the prevention of graft rejection.

In HIV-affected patients, with incomplete or without antiretroviral treatment, HIV infection leads to low CD4 + T cells count, and the decrease in these cells gives rise to opportunistic diseases (Chinen and Shearer, 2010).

Autoimmune diseases are a heterogenous group of diseases characterized by loss of tolerance to self-antigens, leading to the development of autoantibodies and activation of the immune system, resulting in immune complex deposits, organ failure, and ultimately death in the most severe cases (Kaul et al., 2016; Denton and Khanna, 2017). Treatment options involve mainly the use of non-specific immunosuppressive agents such as high-dose corticosteroids or cyclophosphamide (alkylating agent), as well as targeted therapies such as rituximab (anti-CD20) and anti-TNFα (infliximab and adalimumab). The increased risk of infectious disease upon immunosuppressive therapies is well documented (Lode and Schmidt-Ioanas, 2005; Barber and Clarke, 2020; Mitratza et al., 2021), but the impact of autoimmune diseases and their therapies on COVID-19 disease course remains debated (Kastritis et al., 2020; Murtas et al., 2020; Pablos et al., 2020; Zen et al., 2020; Zhong et al., 2020; Liu et al., 2021b).

## Study Population

Our literature review found 44 patients with prolonged SARS-CoV-2 infection affected with secondary immunodeficiency, summarized in Table 1. A more detailed version of Table 1 is attached in Supplementary Table 1. These patients were described in papers found using the two search engines: PubMed and Google Scholar with key phrases “SARS-CoV-2 chronic infection,” “SARS-CoV-2 evolution immunocompromised,” and “SARS-CoV-2 evolution immunodeficiency” queried until May 08 2022.

**TABLE 1:** Secondary immunodeficient patient population.

Patient data				Timeline and outcome		Anti-Spike mAb			Antivirals		Ig and plasma			References
					
N°	Age	Sex	Medical conditions	End-point (days)	Outcome (cause of death if not COVID)	BAM	ETE	CAS – IMD	REM	L-R	IV Ig	CP	HP	Study
P1	71	F	CLL	105	R						x	x		Avanzato et al., 2020
P2	75	M	CLL	197	R				x			x		Monrad et al., 2021
P3	late 60s	M	CLL	91	R	x								Jensen et al., 2021
P4	72	M	CLL	61	R	x			x			x		Truffot et al., 2021
P5	76	F	CLL	72	R				x			x		Martinot et al., 2021
P6	68	M	CLL	43	R	x			x		x	x		Bronstein et al., 2021
P7	23	M	ALL	410	R			x	x			x		Bailly et al., 2021
P8	3	F	ALL	91	R									Truong et al., 2021
P9	21	M	ALL	45	R				x			x		Truong et al., 2021
P10	2	M	ALL	51	R				x					Truong et al., 2021
P11	21	F	ALL	98	D				x					Leung et al., 2022
P12	55	F	AML	42	R	x			x					Lohr et al., 2021
P13	Early 60s	M	FL	103	R	x						x		Jensen et al., 2021
P14	52	M	FL	194	D				x	x			x	Pérez-Lago et al., 2021
P15	47	M	FL	120	R				x	x	x		x	Pérez-Lago et al., 2021
P16	63	F	FL	69	D				x	x	x		x	Pérez-Lago et al., 2021
P17	52	F	FL	100	R						x			Lynch et al., 2021
P18	Unkn	F	FL	165	R									Mancon et al., 2022
P19	61	F	DLBCL	58	R				x					Borges et al., 2021
P20	48	F	DLBCL	335	R				x		x	x		Nussenblatt et al., 2022
P21	70	F	NHL	292	R			x	x		x			Gandhi et al., 2022
P22	70	M	MBCL	102	D				x			x		Kemp et al., 2021
P23	60	M	MCL	39	R				x			x		Baang et al., 2021
P24	33	M	HL	45	R but still PCR +	x								Bronstein et al., 2021
P25	63	F	CTCL	40	R but still PCR +	x	x							Guigon et al., 2021
P26	73	M	Multiple myeloma	74	D				x			x		Hensley et al., 2021
P27	73	M	Cholangio-carcinoma	21	D	x	x							Focosi et al., 2021b
P28	Early 50s	M	Kidney transplant	64	R							x		Chen L. et al., 2021
P29	Late 60s	M	Heart transplant	40	R	x								Jensen et al., 2021
P30	Mid 60s	F	Kidney transplant	26	R	x						x		Jensen et al., 2021
P31	58	M	Kidney transplant	189	R				x					Weigang et al., 2021
P32	Early 40s	F	AIDS (HIV-Toxo)	32	R	x			x			x		Jensen et al., 2021
P33	66	M	AIDS (HIV-LEMP)	Unkn.	Unkn – probable D (LEMP)									Tarhini et al., 2021
P34	28	M	AIDS (HIV-P. jiroveci-M. avium)	103	R									Álvarez et al., 2022
P35	Late 30s	F	HIV	216	R but still PCR +									Cele et al., 2022
P36	61	F	HIV	93	R									Hoffman et al., 2021
P37	45	M	APL syndrome	154	D			x	x		x			Choi et al., 2020
P38	Early 70s	M	AAV	20	D	x						x		Jensen et al., 2021
P39	87	M	PAOD, Diabetes, HBP, CHD, CKD	27	R	x								Peiffer-Smadja et al., 2021
P40	35	M	Diabetes, HBP, CKD, RVD, JIA	38	R	x								Peiffer-Smadja et al., 2021
P41	61	M	Stroke, PAOD, Diabetes, HBP, CHD, CKD	18	R	x								Peiffer-Smadja et al., 2021
P42	97	M	Dementia, HBP and Diabetes	37	D (decubitus complications)	x								Peiffer-Smadja et al., 2021
P43	64	M	Stroke, Diabetes, HBP, CHD (heart transplant)	48	R	x								Peiffer-Smadja et al., 2021
P44	66	M	Stroke, Diabetes, HBP, RA, CKD (kidney transplant)	45–50	D	x								Peiffer-Smadja et al., 2021

*x, Treatment used; mAb, monoclonal antibody; N°, patient number; F, female; M, male; CLL, chronic lymphocytic leukemia; ALL, acute lymphoblastic leukemia; AML, acute myeloid leukemia; FL, follicular lymphoma; DLBCL, diffuse large B cell lymphoma; MBCL, marginal B cell lymphoma; MCL, mantle cell lymphoma; NHL, non-HL; HL, Hodgkin’s lymphoma; CTCL, cutaneous T cell lymphoma; AIDS, acquired immunodeficiency syndrome; HIV, human immunodeficiency virus; LEMP, progressive multifocal leukoencephalopathy; Toxo, toxoplasmosis; P. jiroveci, Pneumocystis jiroveci; M. avium, Mycobacterium avium; APL, antiphospholipid; AAV, ANCA (anti-neutrophil cytoplasmic antibodies) associated vasculitis; PAOD, peripheral arterial occlusive disease; HBP, high blood pressure; CHD, coronary heart disease; CKD, chronic kidney disease; RVD, restrictive ventilatory disorder; JIA, juvenile idiopathic arthritis; RA, rheumatoid arthritis; Unkn, unknown; R, recovery; D, death; diag, diagnosis; BAM, bamlanivimab; ETE, etesevimab; CAS-IMD, casirivimab – imdevimab; REM, remdesivir; Lopi-Rito, lopinavir-ritonavir; IV Ig, intravenous immunoglobulins; CP, convalescent plasma; HP, hyperimmune plasma. “Endpoint (days)” refers to the time frame between the earliest positive sample of the patient that confirms the diagnosis and the most recent sample available before recovery, death, or discharge.*

Among this population, 27 patients were affected with cancer, one with cholangiocarcinoma (P27) and 26 with hematopoietic malignancies. These encompass chronic lymphocytic leukemias (P1–6), acute leukemias (P7–12), lymphomas (P13–25), and multiple myeloma (P26). All these patients presented with a humoral deficiency, either due to the initial pathology or received treatments that combine anti-CD20 monoclonal antibodies that deplete B cells. This wide spectrum of antibody and chemotherapy regimens, including, for example, bendamustine or cyclophosphamide, has a broad effect on innate and adaptive immune responses. Four patients (P28–P31) were solid organ recipients with drug-induced immunosuppression designed to prevent graft rejection using a wide spectrum of immunosuppressors, such as mycophenolate mofetil, tacrolimus, cyclosporine, azathioprine, and steroids. Five patients (P32–P36) were HIV-infected individuals with CD4^+^ T cells impairment due to the viral infection. Two patients (P37 and P38) were affected with autoimmunity: one with antiphospholipid syndrome and one with ANCA-associated vasculitis. Finally, six patients (P39–P44) were immunocompromised by other associated comorbidities such as diabetes, chronic heart, or kidney disease. Of note, two of these patients (P40 and P44) were affected by autoimmune diseases (juvenile idiopathic arthritis and rheumatoid arthritis, respectively). (No information on an eventual immunosuppressive regimen was recorded for those two patients).

Among the 44 patients, 38 received treatment directly targeting the virus (associated or not to treatment for the cytokine storm or host-based therapies for immunomodulation). Notably, twenty-one (21) patients received anti-spike monoclonal antibodies (mAb) that target the receptor-binding domain (RBD) of the S protein (Kumar S. et al., 2021). Three patients (P7, P21, and P37) received an association of 2 mAb, casirivimab–imdevimab (Regeneron/Roche REGEN-COV/Ronapreve^®^), and 18 patients received bamlanivimab (16 in monotherapy, two in association with etesevimab, both from Lilly).

Of note, 21 patients received direct antiviral treatments; 21 patients were associated with remdesivir (an adenosine analog that directly inhibits the viral RNA polymerase, Gilead), and 3 patients were associated with lopinavir-ritonavir (HIV-1 protease inhibitors that bind to the catalytic site of the protease and impair virion production). Sixteen patients received convalescent plasma (CP), three patients in association with polyvalent immunoglobulins. Eight patients received polyvalent immunoglobulins intravenously [three patients with CP as mentioned, two patients with hyperimmune plasma (HP), and three patients in monotherapies]. Three patients received hyperimmune plasma (HP), two with IV immunoglobulins and one in monotherapy.

All these therapeutic data are summarized in Table 1, and previous immunosuppressive regimen, cytokine storm treatment, and host-based therapies are described in Supplementary Table 1.

Furthermore, viral genomes of patients were analyzed for predicted novel amino acid changes found in the different proteins. We defined novel amino acid changes as those detected as different from the earliest viral sequence obtained from the patient at admission. The underlying variations discussed are based on recurrence and/or structural significance, if reported in other studies. To have a common nomenclature for the corresponding amino acid changes, the ORF1ab polyprotein amino acid changes provided in some papers were converted to the corresponding Nsps. First, the amino acid sequence of the Nsp of interest was aligned with the sequence of polyprotein 1ab using BLAST. Second, the corresponding position of the amino acid change was determined and manually annotated. When only nucleotide sequences were available, the nucleotide changes in the different codons of different genes were manually determined. When our compiled studies showed amino acid changes with respect to the ORF1b gene only, we added nine amino acids to the position of the change to account for the ribosomal slippage in which the first nine amino acids are read in ORF1a and the following amino acids in the ORF1b (Bhatt et al., 2021).

**FIGURE 3:**
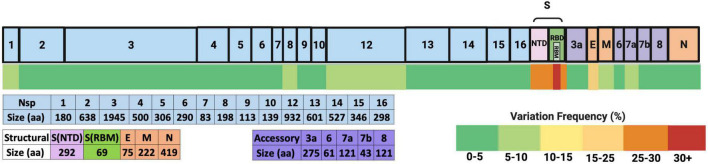
Frequency of amino acid variation in SARS-CoV-2 proteins from the analyzed secondary immunodeficient patients. The SARS-CoV-2 proteins are depicted on top, where the light blue boxes represent non-structural proteins (Nsps) generated from polyprotein 1ab (pp1ab). The NTD and RBM spike domains are shown as pink and green boxes, and other structural proteins are depicted in orange boxes (E, envelope; M, membrane; N, nucleocapsid). The accessory proteins are shown in purple. The total number of amino acids of each protein is depicted below. The heat map scale is shown on the bottom right. The changes shown were isolated from a total of 148 full-length SARS-CoV-2 genomic sequences from 21 patients (shown in S2).

In addition, in all patients examined (Table 1), the number of amino acid changes occurring in each SARS-CoV-2 protein reported by studies that performed full-length genome sequencing of SARS-CoV-2 was aggregated and divided by the number of amino acids per respective protein to generate a variation frequency and allow us to better visualize which proteins are the most changed (Figure 3). The full list of these variations is shown in Supplementary Table 2. To compare with the total number of SARS-CoV-2 sequenced viruses, the percentage of each amino acid change was calculated from the GISAID database as of May 18 2022 (using 10,900,892 as the total number of deposited sequences, and shown in Supplementary Table 3). The subsequent sections focus on the proteins with the most substitutions (in red-dark yellow heat map color on Figure 3) or substitutions with functional significance.

From these data, it could be deduced that most variations occur in the spike protein, more particularly in the receptor-binding motif (RBM) and the NTD. Of note, the E protein has the second highest frequency, followed by Nsp12, Nsp1, Nsp8, ORF7a, and M. Variations in these proteins and others that are either recurrent and/or with functional significance are discussed in the following paragraphs.

## Spike Protein Variations

**FIGURE 4:**
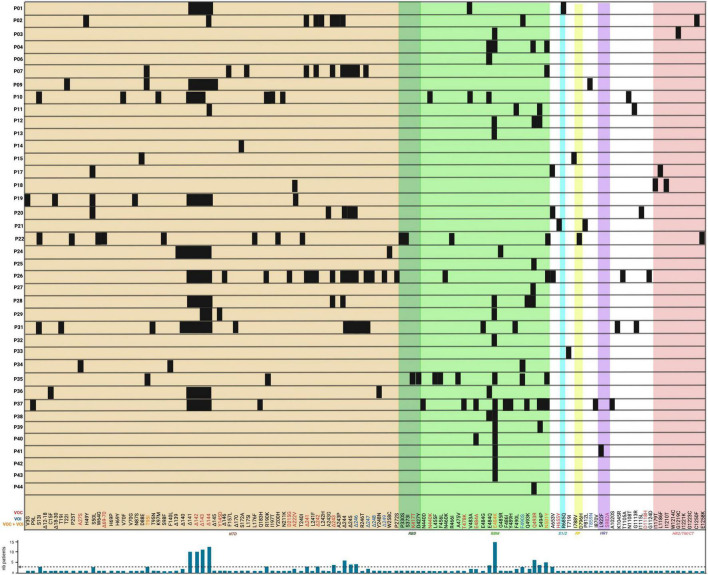
Location of novel SARS-CoV-2 amino acid changes in the spike protein emerging in some immunocompromised patients during chronic infection. The variations are shown as black boxes and represent either amino acid substitutions or deletion with their corresponding identity at the bottom. The different colored areas around the boxes are representative of the spike protein domains corresponding to those shown in Figure 1B: the brown area corresponds to the NTD; the dark green corresponds to the RBD; the pale green corresponds to the RBM of the RBD; the cyan corresponds to the S1/2; the yellow corresponds to the FP; the purple corresponds to the HR1, and the pale red corresponds to either HR2, TM, or CT domains. The amino acid change commonly found only in variants of concern (VOC) are in red font; those only in variants of interest (VOI) are in blue and those found in both VOC and VOI are in orange. Patients P5, P8, P16, P23, P30 did not present any novel amino acid changes in the spike protein and thus are not shown. A histogram depicts the occurrence of variations in the number of patients (shown on *y*-axis as “nb patients”) with the threshold of selection of three patients depicted by a dashed line.

Spike proteins had the highest number of amino acid changes, mainly in the RBM and in the NTD. Interestingly, the emergence of the E484K amino acid substitution (AAS) was observed in 43% of the patients studied (P3, P4, P12, P13, P28, P29, P32, P35, P37, P38, P39, P40, P41, P42, and P43). Secondary immunodeficient patients treated with bamlanivimab showcased a potential example of antiviral-induced selective pressure. Indeed, in April 2021, the emergency authorization use license of bamlanivimab monotherapy was revoked in the United States due to concerns about inefficiencies, as most circulating variants, especially the dominant delta variant, were resistant to neutralization (Gottlieb et al., 2021). Soon after, in January 2022, combined therapy of bamlanivimab and etesivimab, as well as casirivimab and imdevimab, was discouraged in the United States due to less neutralization activity against the now dominant omicron variant (Cavazzoni, 2022). All variations detected in SARS-CoV-2 spike proteins in the 44 patients are shown in Figure 4.

## Variations in the Receptor-Binding Motif

**FIGURE 5:**
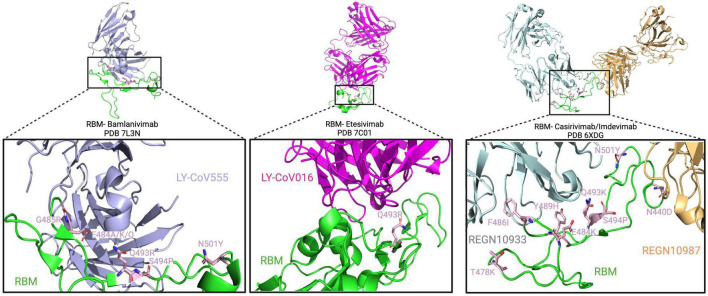
Spike RBM amino acid substitutions in patients treated with monoclonal antibodies. The interaction between the RBM and bamlanivimab, etesivimab, and casirivimab/imdevimab (light blue, pink, gray/yellow) is depicted on top from right to left, respectively. Below, a close-up view of the RBM/antibody interaction is shown with substitutions shown in light pink.

The spike RBM is a 69 amino acid motif (aa 438–506) involved in binding to the host cell receptor. From the selected patients, a total of 19 amino acid changes were found, with the most frequent change being the E484K substitution which was noted in 15 out of 44 patients (P3, P4, P12, P13, P28, P29, P32, P35, P37, P38, P39, P40, P41, P42, and P43). From these patients, 13 out of these 15 (29.5% of total patients) had received monoclonal antibody therapy, and of those, 12 (27% of total patients) were treated with bamlanivimab (Table 1). Furthermore, other variations at the same amino acid position (484) have also been reported. This includes the E484G, E484A, and E484Q substitutions that occurred in 1/44 (P31 – 2.3% of total patients), 2/44 (P37 and P40 – 4.5% of total patients), and 5/44 (P4, P6, P10, P36, and P38 – 11.4% of total patients) patients, respectively. For E484Q, 3 of 5 patients (6.8% of total patients) with this substitution received bamlanivimab, and both patients (4.5% of total patients) with E484A had received casirivimab and bamlanivimab, respectively, but P31 did not receive any monoclonal therapy. In recent studies, Jangra et al. (2021) showed that recombinant SARS-CoV-2 virus harboring the E484K AAS reduced *in vitro* antibody neutralization of human convalescent and post-vaccination sera relative to control virus without this variation. This result was also confirmed by Collier et al. (2021), showing loss of neutralizing activity by vaccine-elicited antibodies and monoclonal antibodies. *In silico* results by Wang et al. (2021) predicted that this AAS could result in favorable electrostatic interactions and tighter binding with the ACE2 receptor. In combination with another change not found in these patients (L452R), it has also been shown that a pseudotyped virus with the E484Q substitution resulted in a reduced neutralization of immune sera from vaccinated (against RBD) non-human primates, convalescent COVID-19 patients, as well as double-dose vaccinated individuals (also against RBD) (Li G. et al., 2021). This result was also confirmed by Ferreira et al. (2021) who also reported a decreased neutralization of pseudotyped virus with both E484Q and L452R alone or in combination using sera of vaccinated individuals. The AAS E484K is observed in beta, gamma, omicron, and mu variants, and E484A is found in the omicron variant (Hodcroft, 2021). Additionally, the 484 residue is in the proximity of suspected bamlanivimab- and casirivimab-binding sites (Figure 5), suggesting that these antibody therapies may have exerted selection pressure.

Another common variant observed in these secondary immunodeficient patients was the Q493R substitution, present in 6/44 (13.6% of total patients) patients (P4, P12, P25, P27, P28, and P44). All of them, except for P28, received bamlanivimab treatment. In combination with bamlanivimab, P25 and P27 also received etesivimab treatment, which binds the RBM in close proximity to this residue (Q493) (Figure 5). At the same position (493), the Q493K substitution was also reported in P28 as well as P37, where the latter had received casirivimab/imdevimab in combination (Choi et al., 2020). In terms of their abundance in the total number of SARS-CoV-2 sequences in GISAID, Q493R and Q493K have a frequency of 30.9 and 0.0079% respectively, suggesting an increased representation of the variation in secondary immunodeficient patients (Elbe and Buckland-Merrett, 2017). Clark et al. (2021) showed that SARS-CoV-2 spike pseudotyped viruses harboring the Q493K substitutions significantly decreased the neutralization of the REGN10933 (Casirivimab) and C1A-VH3-53 antibodies. This decreased neutralization was also noted in the case of Q493R pseudotyped virus (Clark et al., 2021). Similarly, a study by Starr Tyler et al. (2021) also investigated the potential of antibody escape mutations and demonstrated the escape of Q493K from the REGN10987 (imdevimab). Another nearby AAS is S494P, which has been observed in 4 patients (P11, P12, P37, and P39 – 9.1% of total patients), and is in 0.15% of all SARS-CoV-2 sequences in GISAID. This change has also led to the neutralization reduction in antibodies from convalescent and post-vaccinated sera, especially in combination with E484K and N501Y substitutions (Alenquer et al., 2021). These data suggest that Q493K and Q493R AAS could contribute to SARS-CoV-2 resistance to anti-spike monoclonal antibodies in immunodeficient patients.

Moreover, the N501Y AAS was also identified to emerge *de novo* in five patients (P4, P7, P22, P35, and P37), three of whom were treated with monoclonals (P4: bamlanivimab; P7 and P37: casirivimab/imdevimab). This AAS seems to be near the casirivimab-binding site to RBM (Figure 5). Furthermore, N501Y has also been determined to be present in alpha, beta, gamma, omicron, and mu variants. Having this AAS increases the binding affinity of spike proteins to human ACE2 as shown by Tian et al. (2021) and Liu et al. (2021a). Moreover, it has been shown that this substitution decreases neutralization by both H00S022 and 10F9 neutralizing monoclonal antibodies and increases the infectivity of pseudotyped virus by 5-fold compared with the 614G variant in HEK293T cells expressing mouse ACE2 (Li Q. et al., 2021). Interestingly, Niu et al. (2021) also noted that pseudotyped virus with the N501Y change effectively infected mouse-ACE2 expressing 293T cells and detected a successful infection of wild-type BALB/c with a SARS-CoV-2 strain bearing the substitution. A recent study by Liu et al. (2021a) reported that *in vivo*, this substitution enhanced viral fitness in intranasally infected hamsters and intra-cage transmissions. This suggests that N501Y may play a role in a potential viral spillover to mice (Huang et al., 2021).

Besides the aforementioned, other RBM changes were also found in the reviewed population, including the N440K (P10), T478K (P37), and F490S (P2, P34, and P35), which are found in the omicron, delta, and lambda variants, respectively. The N440K variant has been reported to evade the REGN10987 antibody (Starr Tyler et al., 2021), while F490S has been shown to allow resistance to vaccine-elicited sera (Kimura et al., 2021). Furthermore, among millions of GISAID sequences, the frequency of F490S, N440K, and T478K AASs is 0.17, 25.9, and 70.9%, respectively. Other AASs have also been identified in casirivimab/imdevimab receiving patients. In Figure 5, we showed the molecular structures and positions of the monoclonal antibodies and the AASs identified in the RBM of the spike. These findings suggest that either therapy (vaccine or antibody therapy) or convergent evolution explains their emergence.

## (N)-Terminal Domain and other Spike Variations

Besides the RBM, spike amino acid changes were identified in other domains, especially in the 292 amino acid NTDs (aa 14–305). Among the NTD variations reported, the most frequent ones occurred in the range of residues between amino acid positions 139 and 146 and this occurred in 13 out of 44 patients (29.5% of the total patients, P1, P2, P9, P10, P11, P19, P24, P26, P28, P29, P31, P36, and P37). The second most common variant was a deletion occurring in a range of residues between amino acid positions 241 and 249 in 6 out of 44 patients (13.6% of total patients, P2, P7, P20, P26, P28, P31). In terms of substitutions, S50L (P17, P19, and P20), T95I (P7, P9, and P35), and R190K (P10, P26, and P35) were identified in 3 patients (6.8%). Moreover, other deletions were also reported at position 69–70 (Δ69–70) for P22, and both patients P19 and P37 had deletions from aa 18 to 30 (Δ18–30) and 12 to 18 (Δ12–18), respectively. Several of these amino acid changes were also present in VOCs and/or VOIs: a spike deletion at position 141 is present in the alpha variant, and the ones spanning from position 142–144 (Δ142–144) are in the omicron variant. Furthermore, the beta variant contains deletions from residue 241 to 243 (Δ241–243), whereas the lambda variant has a deletion from position 246 to 249 (Δ246–249). Both alpha and omicron variants contain the Δ69–70 deletion, and the lambda variant contains the T19I substitution (Hodcroft, 2021). Functionally, Mccarthy et al. (2021) showed that the combination of deletions (Δ69–70 and Δ 144/145), (Δ141–144, Δ144/145, and Δ146), and Δ243–244 all abolished binding to the 4A8 neutralizing antibody, indicating these regions in the NTD to be possible immunodominant epitopes for neutralization. Such an effect was also tested by Graham et al. (2021) where a significant reduction of neutralization by NTD targeting antibodies was also noted in pseudoviruses with the Δ141 spike deletion in combination with the D614G substitution. Furthermore, the researchers studying the chronic infection of P19 showed that double deletion Δ69–70, and another substitution reported in the spike fusion peptide D796H decreased the sensitivity to convalescent plasma *in vitro* (Kemp et al., 2021). It was also revealed that the Δ69–70 deletion had higher infectivity than a wild-type SARS-CoV-2 and that D796H was the main contributor to escaping neutralization while showing reduced infectivity (Kemp et al., 2021). The mutations seen in the NTD are functional mutations that overlap domains and have been observed in both VOCs and VOIs, and thus are worthy of significant focused interest for surveillance of future variants with altered biology. Besides the NTD and RBD, other amino acid changes occur in the spike such as the recurrent S13I in three patients (7.5% of total patients, P10, P22, and P31), the T859N (P9), and D1118H (P31) substitutions (2.5% of total patients). The T859N is also found in the lambda variant, and the D1118H is found in the alpha variant. The significance of these changes has not yet been determined. In sum, these results encompass the most recurrent amino acid changes observed in the spike protein of secondary immunodeficient patients described in the literature.

## Variations in Non-Spike Proteins

### Envelope

**FIGURE 6:**
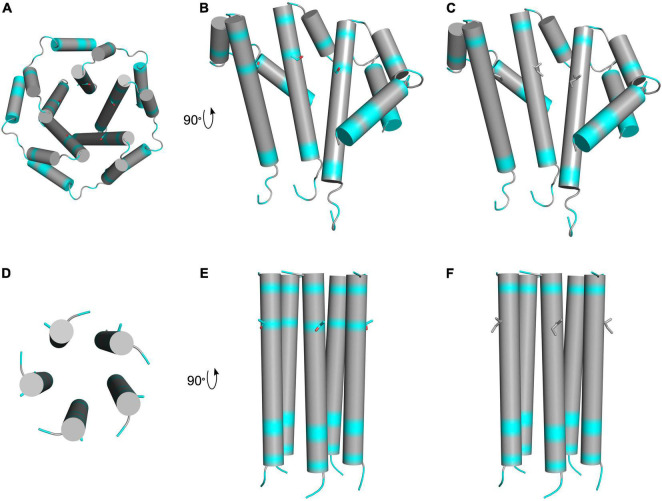
Schematic presentation of hydrophobic regions of T30I mutant E protein. The top row depicts LMPG micelle solution NMR structures of truncated SARS-CoV-1 E protein residues 8–65 in homopentameric channel complex (PDB: 5 × 29). Hydrophobic residues are colored in gray, and hydrophilic residues are colored in cyan. Oxygen atoms are colored in red. **(A)** Schematic view of the pore formed by the wild-type protein complex showing threonine 30 side chains. **(B)** Three subunits of the wild-type complex showing threonine 30 side chains. **(C)** Three subunits of the T30I mutant complex showing isoleucine 30 side chains. The bottom row depicts solid-state NMR structures of SARS-CoV-2 E protein transmembrane domain in homopentameric channel complex (PDB: 7K3G). Hydrophobic residues are colored in gray, and hydrophilic residues are colored in cyan. Oxygen atoms are colored in red. **(D)** View of the pore formed by the wild-type protein complex showing threonine 30 side chains. **(E)** Wild-type complex showing threonine 30 side chains. **(F)** T30I mutant complex showing isoleucine 30 side chains.

The SARS-CoV-2 envelope (E) is a 75 amino acid hydrophobic transmembrane protein that is crucial for infecting host cells (Boson et al., 2021). It is composed of three domains including the N-terminal domain (NTD; aa 1–8), transmembrane domain (TM; aa 9–38), and the C-terminal domain (CTD; aa 39–75) (Mandala et al., 2020). In other coronaviruses, it is thought that the TM acts as an ion channel and that the CTD interacts with other proteins like cellular adapters (Schoeman and Fielding, 2019). From our analysis of patients, all five AASs in the E protein were located in the TM and the CTD. From those, 9 out of 44 reviewed patients (20.4% of total patients) presented the T30I AAS (P10, P11, P15, P17, P18, P19, P20, P22, and P37) which is found in the transmembrane domain of this protein. A search of the 10,900,892 SARS-CoV-2 sequences recorded by GISAID, as of May 18 2022, indicated that this very rare variation is only found in 1,156 sequences (0.011%) (Elbe and Buckland-Merrett, 2017). Using FoldX, one study predicted that this change could be a stabilizing substitution (Rahman et al., 2021). To gain more insight on its structural effects, we modeled the T30I AAS into previously determined NMR structures of the SARS-CoV-1 (PDB: 5X29) and SARS-CoV-2 (PDB: 7K3G) envelope protein (Figure 6). Despite sequence similarity, there are notable differences between the structures, among them the positions of residue 30. In the 5X29 structure, Thr30 is in an interhelical position, whereas this residue is in a lipid-facing position in the 7K3G structure. It is unclear if these variations result from differing experimental techniques or simply plasticity of the protein complex. Due to the ambiguous position of Thr30, we additionally generated models using DeepMind AlphaFold 2. However, these models also suffered from inconsistent Thr30 positions, and thus, the precise position of this residue is uncertain. Nonetheless, in both the interhelical and lipid-facing positions, the T30I AAS increases the hydrophobicity of the transmembrane domain. The substitution from threonine, a hydrophilic amino acid, to isoleucine, a hydrophobic amino acid, would likely have a stabilizing effect, as the surrounding residues and lipid environment are also hydrophobic. Although the function of this precise change in SARS-CoV-2 is unknown, Nieto-Torres et al. (2014) have investigated the ion channel activity of the E protein in *in vitro* and *in vivo* pathogenesis of SARS-CoV; interestingly, they observed a lesser disease severity in mice infected with viruses lacking ion-channel (IC) activity, as opposed to those infected with viruses lacking IC activity with the T30I AAS, suggesting an impact on the presentation of the SARS-CoV-2 pathogenesis. The E protein can be sensed by TLR2-dependent host cell signaling to produce proinflammatory cytokines (Tasakis et al., 2021), suggesting that variations may have multiple effects on ion conductivity, pathogenesis, and inflammation.

Besides T30I, five other AASs in the E protein were reported in the studied population, including the N48D and S50I (P10), T9I (P18), L19I (18), and L21F (P23) but as of yet, none has been reported to have an impact on the TM and CTD domains. Furthermore, as of May 18 2022, the only variation detected in the TM of E is the T9I substitution, with a variation frequency of 33.5% in 10,900,892 GISAID sequences, present in VOCs (Hodcroft, 2021). From our analysis, it could be speculated that the T30I AAS could perhaps be selected in immunodeficient settings, but more research on SARS-CoV-2 E AASs is needed to clarify whether this is the case.

### Membrane Protein

The coronaviral membrane (M) is a 222 amino acid protein known to play a role in virion assembly and morphogenesis, among other processes (Liu et al., 2007; Jörrißen et al., 2021). In studied patients, the most common AAS identified was the H125Y, in five patients (11.4% – P10, P17, P20, P22, and P33). In addition, other AASs such as the A2S AAS were identified in 2 patients (4.5% – P1, P37). Yet, no functional impact has been reported from the literature, and the variation frequency of both H125Y and A2S in the total GISAID sequences is 0.11 and 0.24%, respectively. However, some defining AASs in the studied immunodeficient patients have been noted in VOCs, particularly in the delta (A82T) and omicron variants (D3G, Q19E, and 63T) (Hodcroft, 2021).

### Nsp1

Nsp1 coronavirus proteins are known to shut down host protein translation to inhibit the expression of key genes involved in viral control (Nakagawa and Makino, 2021). As an 180 amino acid protein, it has been predicted to be made of an NTD (aa 1–128), a linker (aa 129–148), and a CTD domain (aa 149–180) (Schubert et al., 2020). The CTD has been shown to inhibit cellular gene expression by binding to the 40S ribosomal entry channel, whereas the NTD allows SARS-CoV-2 mRNA to escape this inhibition by binding to its leader sequence and stabilizing its interaction with the ribosome (Mendez et al., 2021).

In the analyzed secondary immunodeficient patients, several AASs and deletions were detected in the Nsp1 protein in patient viruses whose genomes were fully sequenced (P1, P2, P9, P10, P16, P17, P19, P21, P22, P28, and P31). From those, there were recurrent changes in the NTD including the amino acid deletion at position 85, which occurred in 2 (4.5%) patients (P1 and P31), and the AASs R124C (P10 and P22) and I114T (P2 and P19), which were also detected in two patients, respectively. The M85 deletion, R124C, and I114T substitutions are, respectively, present in 1.78, 0.14, and 0.05% of total GISAID sequences.

Structurally, the Nsp1 deletion at amino acid 85 has been previously shown to lead to a lower type I interferon response in infected Calu-3 cells, in contrast to wild-type Nsp1 (Lin et al., 2021). In the case of the AAS R124C, Mou et al. (2021) previously predicted *in silico* that this SNP has a destabilizing effect on the Nsp1 protein structure. Later, an *in vitro* study by Mendez et al. (2021) found that the R124A amino acid change at the same position along with the K125A AAS promotes host RNA decay, reduces host mRNA translation levels by destabilizing the binding to the 40S ribosomal subunit, and reduces the repression of SARS-CoV-2 leader containing transcripts. Moreover, Kim et al. (2021) also showed that the R124A/K125A changes did not have any effect on the levels of caspase-1 proteins *in vitro*, in contrast to the wild-type Nsp1, which significantly reduced caspase-1 levels and blocked its cleavage.

In summary, we identified recurrent Nsp1 variations in the NTD domain that could be involved in interfering with the host defenses. It would be of interest to investigate if there is a selection of Nsp1 NTD variations in immunodeficient individuals.

### Nsp3

Nsp3 is the largest multi-domain coronaviral protein with a total of 1,945 amino acids (Lei et al., 2018; Gruca et al., 2021). It is involved in the proteolytic cleavage of polyproteins pp1a and pp1ab and the removal of K18-linked polyubiquitin and interferon-stimulated gene 15 (ISG15) from cellular proteins (Lei et al., 2018; Armstrong et al., 2021). From its many domains, its protease activity is conferred by the papain-like protease domain (aa 813–1076) (Armstrong et al., 2021). In this domain, two recurrent AASs were identified in the studied patients including T820I (4.5% of total patients, P11 and P23) and P822L (4.5%, P15 and P23). These occur with a frequency of 0.04 and 3.8% in the total GISAID sequences, and their impact on the role of Nsp3 is not yet studied. Other changes were also identified (S2), with the P1228L AAS (P7) being present in 35.6% of total GISAID sequences. This falls in the α-helical loop (aa 1,177–1,333), which is not yet well characterized. Therefore, it would be of great interest to investigate the role of the identified variations in Nsp3 and their possible impact on its catalytic activity.

### Nsp6

Nsp6 is a transmembrane protein that is not very well characterized in SARS-CoV-2 infection (Kumar A. et al., 2021). Sun et al. (2022) showed that this protein can target the ATPase proton pump component involved in lysosomal acidification, ATP6AP1, to trigger NLRP3-dependent pyroptosis in lung epithelial cells. In the reviewed patients, the L37F protein-coding change was noted to emerge *de novo* in three different cases (11.4% of total patients, P10, P17, P20, P21, P31), and this AAS is part of around 2.0% of GISAID sequences (Lynch et al., 2021; Truong et al., 2021; Weigang et al., 2021). Recently, Benvenuto et al. (2020) predicted that such an amino acid change led to a lower stability of the Nsp6 protein structure and suggested a role of Nsp6 in binding with the ER. This AAS was also studied by Wang R. et al. (2020) who analyzed around 76,000 sequences in GISAID up to 19 October 2020, and correlated it with lower death ratios and transmission rates. Through bioinformatic analysis, it was also shown to be destabilizing and less functional compared with the wild-type (Wang R. et al., 2020). Furthermore, this same AAS has also been observed to weaken the interaction of Nsp6 with ATP6AP1, thus reducing lysosome acidification and pyroptosis induction (Sun et al., 2022). These observations stress the importance of the L37F AAS in both immunodeficient and immunocompetent individuals, given the relatively high variation rate in the reviewed patients and in GISAID, which might suggest an unappreciated fitness benefit conferred by this variation. The study of patients with chronic SARS-CoV-2 infection would be another source of data for predicting the transmissibility and lethality of SARS-CoV-2, especially in the context of immunodeficiency.

### RdRp/Nsp12

The RNA-dependent RNA polymerase (RdRp) involved in SARS-CoV-2 genome replication and transcription of genes is composed of a catalytic subunit known as Nsp12 as well as two accessory subunits, Nsp8 and Nsp7 (Gao et al., 2020b). The Nsp12 domain resembles a right hand, comprising the fingers subdomain, which interact with the template strand RNA and direct it into the active site, and the palm domain, which forms the catalytic active center. The RdRp is the target for antiviral drugs such as remdesivir (RDV and GS-5734), which can incorporate itself in the nascent viral RNA chains, causing premature transcriptional termination (Warren et al., 2016). In the study population, many RdRp variations were identified in patients who were treated with remdesivir (nine patients: 20.4%, P2, P5, P9, P14, P16, P21, P22, P23, and P31). From these, a few AASs were identified in the RdRp palm domain including V792I (5%, P16 and P22), E796D (P9), C799R (P14), and E802D, respectively. In the whole GISAID database, these occur at a frequency of less than 0.0025%. Interestingly, an *in vitro* study by Szemiel et al. (2021) showed that a palm domain substitution in a conserved residue (E802D) of the RdRp decreases the sensitivity to remdesivir and viral fitness in a competition assay; this same amino acid change (E802D) was found in P21, decreased binding to remdesivir, and has a fitness cost (Gandhi et al., 2022). As this substitution was found to be close to residues involved in binding with nascent RNA (aa 813–815), it was suggested that the RdRp could have structural changes that could allow elongation of template RNA, even when remdesivir is incorporated (Szemiel et al., 2021). Indeed, this highlights those substitutions in the RdRp finger, and palm domain should be studied more carefully to determine if these play a role in conferring resistance to antivirals against the RdRp.

### Nsp13

The helicase protein (Nsp13) plays a role in unwinding duplex RNA and DNA in a 5′ to 3′ direction (Vazquez et al., 2021). In the patients we studied, the D374E substitution identified in P22 is an AAS that occurs in one of the residues identified in the NTP hydrolysis active site, and its functional effect is yet to be determined (Jia et al., 2019). Although this AAS is very rare (9/10,900,892 GISAID sequences), since this Nsp13 is one of the proteins involved in the RTC formation, it would be of interest to investigate such changes and their role in viral replication.

### ORF3a

The SARS-CoV-2 ORF3a is an integral membrane protein that has been shown to play a role in inducing apoptosis (Ren et al., 2020) in infected cells, in promoting lysosomal exocytosis (Chen D. et al., 2021), and in blocking the formation of autolysosomes (Miao et al., 2021; Qu et al., 2021). It has also been shown to inhibit STAT1 phosphorylation *in vitro* (Xia et al., 2020). In the described patients, nine variations were identified including the S171L (P9, 0.69% GISAID frequency). Chen D. et al. (2021) showed that the SARS-CoV-2 ORF3a S171E AAS abolished the production of *trans*-soluble *N*-ethylmaleimide-sensitive factor (NSF) attachment protein receptor (SNARE) complex proteins involved in fusing the lysosome to plasma membranes; additionally, the amino acid substitution abolished the ability of ORF3a to increase the Ca^2+^ levels in the cytoplasm. Furthermore, the S171L substitution was predicted *in silico* to increase protein instability with a turn structure replaced by a coiled coil (Azad and Khan, 2021), suggesting that it could be a functional mutation selected in patients.

### ORF7a

The ORF7a accessory protein is believed to play a role in modulating host immune responses (Redondo et al., 2021). In the case of the 44 patients described here, the AASs S81P occurred twice in P14 and P22 (4.5%), and the A105V, which occurred in P9, has been characterized before. In a 62-patient Romanian cohort, Lobiuc et al. (2021) showed that 27.5% were infected with the A105V AAS, and they experienced a twofold higher death rate than others without A105V. The researchers then did a bioinformatic analysis of this change and predicted an increased stability by allosteric effects (Lobiuc et al., 2021).

In summary, we have identified many recurrent SARS-CoV-2 amino acid changes to emerge *de novo* in immunodeficient patients in a variety of proteins that have previously been identified to have a structural effect. This could be the result of different host selection pressures as some proteins (E, Nsp1, M, and ORF7a) had a relatively higher frequency compared with others (Figure 3). More investigation and a bigger study population are needed to make a definitive conclusion.

## Discussion

Prolonged infections resulting from weakened impaired immune responses allow the virus to persist, providing opportunities for increased viral replications and accumulations of mutations, some of which may be novel (Avanzato et al., 2020; Monrad et al., 2021). Thus, as the COVID-19 pandemic continues, it is crucial to track mutations arising in circulating and novel strains that can potentially become VOCs and VOIs to help predict their role in transmission and pathogenesis.

Case studies of chronically infected individuals with immunodeficiency could help gain insights into how the virus evolves in such settings. In this review, we highlighted 44 patients with secondary immunodeficiencies that were chronically infected with SARS-CoV-2 and received a variety of treatments, some of which may exert a selective pressure on the virus (De Vlaminck et al., 2013) (e.g., antiviral drugs targeting a specific protein site). Early studies showed that treatments with monoclonal antibodies should be used with great caution as they have been demonstrated to exert selective pressures on viruses. Focosi et al. (2021a) reviewed case series and reports and observed frequent emergence of single-nucleotide changes in the RBD regions of the spike gene when under the pressure of monoclonal antibodies; additionally, they noted that the mutational pressure from convalescent plasma was different in nature with deletions being more present, presumably due to the polyclonal nature of the antibodies. Alternatively, polyclonal antibodies recognizing different spike epitopes or combination therapy could be used to reduce selection pressure and treatment resistance. Efforts are being made to design broadly neutralizing SARS-CoV-2 and pan-coronavirus antibodies, some relying on the principle of targeting conserved regions that have a high fitness cost if altered (Cameroni et al., 2021; Martinez et al., 2021; Saunders et al., 2021; Shrestha et al., 2021; Tan et al., 2021). Such consideration and strategies when designing new therapies are needed to deliver therapeutics with longevity for the use of the current pandemic and future ones that will inevitably arise.

A careful approach is required in administering future antivirals targeting a specific site of SARS-CoV-2 proteins, especially as novel therapies such as molnupiravir, which targets the RdRp (Fischer et al., 2021), and paxlovid, which targets the SARS-CoV-2 main protease (Nsp5) by reacting reversibly with a cysteine residue at its active site (Pavan et al., 2021), are slated for approval. If these antivirals do not have a high genetic barrier for mutational escape, lengthy efforts and enormous resource commitments could be wasted on therapies that the world has already begun to hail as an end to the pandemic.

Besides the spike protein, it was interesting to find a recurrent AAS T30I in the E protein transmembrane motif in 9 out of 44 different patients (20.4% of total patients) who presented the T30I AAS (P10, P11, P15, P17, P18, P19, P20, P22, and P37). Like the latter, other variations were recurrent in the reviewed patients with a frequency greater than those found in the GISAID database. This was the case of the M protein A2S (4.5% of total patients, 0.24% of GISAID) and H125Y (11.4% of total patients, 0.11% of GISAID) AASs, as well as the Nsp1 R124C (4.5% of total patients, 0.14% of GISAID) and I114T (4.5% of total patients, 0.05% of GISAID), among others. One explanation for this difference could be that these mutations are specific to an immunodeficient environment, where certain immune selective pressures could be different, e.g., weakened. On the contrary, other AASs present in circulating VOCs were noted to emerge in the reviewed patients such as the Nsp3 P1228L (35.6% of GISAID), which could reflect the adaptation of the virus to the human host; however, the functional effect of such variations remains to be elucidated in further studies.

Although of interest, this literature review of SARS-CoV-2 variations in immunocompromised patients has limitations. Previous immunosuppressive regimens were not always known in detail, and this could lead to incomplete evaluation of the extent of immunodeficiency in the studied population. Another confounding factor is the variation in the standard of care for SARS-CoV-2 infection throughout the pandemic. Indeed, variation in treatment regimens over time makes the rise of mutations difficult to interpret, especially considering the variable time from treatment to the moment of infection. Moreover, virus sequencing is often from samples originating from the nasopharynx, which is part of the upper respiratory tract. These samples are not necessarily representative of the virus composition in the lower respiratory tract. Furthermore, the virus replicating in the lower respiratory tract may experience different selection pressures than the upper respiratory tract. In addition, the GISAID frequencies obtained are derived from uploaded consensus sequences in contrast to our patients’ sequencing data that have variable consensus agreement; therefore, the AASs we note may be more noisy and non-selective compared with the GISAID AASs. GISAID frequencies can also include immunocompromised individuals although we expect these to be in the minority. Of note, in the 44 patients that we compiled, all viruses were not sequenced at the beginning of SARS-CoV-2 infection or sequenced at the same timepoint during the disease, once again leading to comparison discrepancies. In addition, some of the papers focus on spike proteins only. Finally, although we chose to focus on secondary immunodeficiencies due to the larger number of patients with analyzed viral genomes, mutations in primary immunodeficient patients (Bucciol et al., 2021; Ciuffreda et al., 2021; Cabañero-Navalon et al., 2022) are slowly being characterized that could add to the topic of viral evolution in the context of immunodeficiencies at large.

## Conclusion

In this review, several variations were found in the spike or Nsp12 proteins, which are important therapeutic targets. We also identified several recurrent variations in E, Nsp1, M, and ORF7a proteins that may play an important role in SARS-CoV-2 pathogenesis. Determining whether these variations emerged through selection in the immunodeficient patients or resulted from the adaptation of SARS-CoV-2 to the human host will require further study. However, the breadth and impact of mutations characterized in patients with secondary immunodeficiency highlight the relevance of monitoring the evolution of SARS-CoV-2 in immunocompromised individuals, not only to identify potentially adaptive novel mutations but also to mitigate the risk of introducing variants that may pose increased health threats to communities.

## Author Contributions

SV and LA: conceptualization and supervision. NM, GG, DP, MH, PN, and AB: writing—original draft preparation. NM, GG, DP, MH, PN, AB, LA, and SV: writing—review and editing. All authors have read and agreed to the published version of the manuscript.

## Conflict of Interest

The authors declare that the research was conducted in the absence of any commercial or financial relationships that could be construed as a potential conflict of interest.

## Publisher’s Note

All claims expressed in this article are solely those of the authors and do not necessarily represent those of their affiliated organizations, or those of the publisher, the editors and the reviewers. Any product that may be evaluated in this article, or claim that may be made by its manufacturer, is not guaranteed or endorsed by the publisher.
